# Supplementary material for: A set of multi-entry identification keys to African frugivorous flies (Diptera, Tephritidae)
Source: Zookeys. 2014 Jul 24;(428):97–108. doi: 10.3897/zookeys.428.7366 (PMC4143993; doi:10.3897/zookeys.428.7366)
Supplement: Supplementary material 6 — Key to Ceratitis [file zookeys-428-097-s006.zip › SF6_ZooKeys_key to Ceratitis/key/SF6_ZooKeys_key to Ceratitis/Media/Html/Ceratitis manjakatompo.htm]

Ceratitis (Ceratitis) manjakatompo Hancock


***Ceratitis*** **(*Ceratitis*) *manjakatompo*
Hancock**

*Ceratitis* (*Ceratitis*) *manjakatompo*
Hancock, 1984: 283.

Body
length: 3.54 (3.5-3.6) mm; wing length: 3.6 (3.55-3.65) mm.

Female

Head.
Antenna yellow-orange. Third antennal segment three times as long as second segment.
Arista with short hairs over entire length, longer than in other *Ceratitis* species
(except *C. cornuta*). Frons pale yellow to yellow; with short scattered
hairs which are largely the same colour as frons, at most slightly darker. All
cephalic bristles dark, except postocellar and outer verticals. Face
yellow-white. Genal bristles dark reddish, strongly developed.

Thorax.
Postpronotum, white with large black spot. Mesonotal pattern: ground colour
orange-brown, microtrichiae pattern silvery with brownish shine, spots
black-brown except conspicuous sutural white spots, prescutellar white markings
merged. Scapular setae pale. Scutellum yellow-white, basally with two dark
separate spots, apically with three merged spots, weakly incised. Basal spots
weakly joined with apical spots. Anepisternum with lower half darker yellow but
not distinct.�

Legs
yellow; setation typical for subgenus, mainly yellow or reddish. Ventral spines
on fore femur reddish.

Wing
bands with markings yellow. Banding; marginal band with clear and complete
interruption; cubital band free; medial band narrow, joined with marginal;
crossvein r-m beyond middle of discal cell. Vein R1 before or equal with
crossvein r-m. Crossvein dm-cu posteriorly more inwards than anteriorly.

Abdomen.
Yellow. Setation and banding typical for subgenus. Oviscape shorter than
abdominal terga.

Male

Unknown.

(Description
after De Meyer, 2000)
